# *Pseudomonas oligotrophica* sp. nov., a Novel Denitrifying Bacterium Possessing Nitrogen Removal Capability Under Low Carbon–Nitrogen Ratio Condition

**DOI:** 10.3389/fmicb.2022.882890

**Published:** 2022-05-20

**Authors:** Mingxia Zhang, Anzhang Li, Qing Yao, Botao Xiao, Honghui Zhu

**Affiliations:** ^1^Guangdong Provincial Key Laboratory of Fermentation and Enzyme Engineering, School Biology and Biological Engineering, South China University of Technology, Guangzhou, China; ^2^Guangdong Provincial Key Laboratory of Microbial Culture Collection and Application, Key Laboratory of Agricultural Microbiomics and Precision Application, Ministry of Agriculture and Rural Affairs, State Key Laboratory of Applied Microbiology Southern China, Guangdong Microbial Culture Collection Center, Institute of Microbiology, Guangdong Academy of Sciences, Guangzhou, China; ^3^Guangdong BOWOTE BioSciTech, Co., Ltd., Zhaoqing, China; ^4^Guangdong Province Key Laboratory of Microbial Signals and Disease Control, College of Horticulture, South China Agricultural University, Guangzhou, China

**Keywords:** *Pseudomonas*, 16S rRNA gene, phylogenetic analysis, genomic analysis, denitrifying bacteria, low carbon–nitrogen ratio

## Abstract

*Pseudomonas* is a large and diverse genus within the *Gammaproteobacteria* known for its important ecological role in the environment. These bacteria exhibit versatile features of which the ability of heterotrophic nitrification and aerobic denitrification can be applied for nitrogen removal from the wastewater. A novel denitrifying bacterium, designated JM10B5a^T^, was isolated from the pond water for juvenile *Litopenaeus vannamei*. The phylogenetic, genomic, physiological, and biochemical analyses illustrated that strain JM10B5a^T^ represented a novel species of the genus *Pseudomonas*, for which the name *Pseudomonas oligotrophica* sp. nov. was proposed. The effects of carbon sources and C/N ratios on denitrification performance of strain JM10B5a^T^ were investigated. In addition, the results revealed that sodium acetate was selected as the optimum carbon source for denitrification of this strain. Besides, strain JM10B5a^T^ could exhibit complete nitrate removal at the low C/N ratio of 3. Genomic analyses revealed that JM10B5a^T^ possessed the functional genes including *napA, narG, nirS, norB*, and *nosZ*, which might participate in the complete denitrification process. Comparative genomic analyses indicated that many genes related to aggregation, utilization of alkylphosphonate and tricarballylate, biosynthesis of cofactors, and vitamins were contained in the genome of strain JM10B5a^T^. These genomic features were indicative of its adaption to various niches. Moreover, strain JM10B5a^T^ harbored the complete operons required for the biosynthesis of vibrioferrin, a siderophore, which might be conducive to the high denitrification efficiency of denitrifying bacterium at low C/N ratio. Our findings demonstrated that the strain JM10B5a^T^ could be a promising candidate for treating wastewater with a low C/N ratio.

## Introduction

*Pseudomonas* is widely distributed in various natural environments including water, soil, sediment, plants, animals, and clinical samples, and this genus contains a large number of species (Zou et al., 2019; Li et al., 2020; Mamtimin et al., 2021). The genus *Pseudomonas* was first proposed with the description of *P. aeruginosa* JCM 5962^T^ by Migula (1894) and belongs to the family *Pseudomonadaceae* within the class *Gammaproteobacteria*. Recently, Lalucat et al. (2021) proposed the division of *Pseudomonas* into five new genera: “*Halopseudomonas*,” “*Linyingimonas*,” “*Stutzerimonas*,” “*Alcaligenimonas*,” and “*Ubiquimonas*” based on the phylogenetic and phylogenomic analyses, and the genus *Halopseudomonas* published by Rudra and Gupta (2021) has been validated. *Pseudomonas* strains displayed some potential roles, such as the high biodegradation capability of polycyclic aromatic hydrocarbons, sulfide oxidation, phosphate accumulation, and denitrification (Xie et al., 2016, 2021; Sun et al., 2019; Zhang R. C. et al., 2020). For example, a denitrifying phosphorus-accumulating bacterium *P. stutzeri* ADP-19 could remove 96.9% of nitrate and 73.3% of phosphorus under aerobic conditions (Li et al., 2021); a heterotrophic nitrifying–aerobic denitrifying bacterium *P. bauzanensis* DN13-1 could remove 98.8, 98.9, and 65.9% of nitrite (${\text{NO}}_{2}^{-}$-N), nitrate (${\text{NO}}_{3}^{-}$-N), and ammonium (${\text{NH}}_{4}^{+}$-N), respectively (Zhang M. et al., 2020). Although the 16S rRNA gene is the basic tool for current bacterial classification, it cannot be used availably to differentiate closely related species of *Pseudomonas* (Wang et al., 2020). Thus, the taxonomy of *Pseudomonas* strains has evolved with the available methodologies. Up-to-date Bacterial Core Gene set (UBCG, 92 bacterial core gene sets commonly present in all bacterial genomes which are shown on the website of EZBioCloud^1^) together with a phylogenomic pipeline could provide the accurate phylogenomic trees for the taxonomic purpose (Na et al., 2018).

Nitrogen pollution mainly comes from the excessive use of fertilizers, poultry production, domestic sewage, industrial manufacture, and aquaculture wastewater (Rezvani et al., 2019). Excess nitrite and nitrate accumulation can cause eutrophication and pose threat to aquatic animals and human health. For instance, excess nitrate could cause the regressive, circulatory, and inflammatory damages in the post-larvae and juvenile of *Macrobrachium amazonicum* (Dutra et al., 2020). Among the nitrate-contaminated wastewaters, the low organic carbon-to-nitrogen (C/N) ratio wastewater is a common type such as the polluted groundwater, industrial wastewater, rural sewage, and effluent from wastewater treatment plants (Deng et al., 2016; Hong et al., 2019; Gao et al., 2020).

Biological denitrification is an effective and general method for reducing nitrate to nitrogen gas due to its high efficiency, low cost, and environmental friendliness (Pang and Wang, 2021). Heterotrophic denitrification, which utilizes the organic carbon sources as electron donors, has a higher denitrification rate compared with autotrophic denitrification (Yang et al., 2020). However, the deficiency of available carbon source is an intractable problem for heterotrophic denitrification in the treatment of wastewater with low C/N ratio. To ensure sufficient electron donors for the denitrification process, the conventional solution is to add external organic carbon (often methanol, ethanol, glucose, and acetic acid) into the wastewater with low C/N ratio (Wang et al., 2018). Nevertheless, the addition of external organic carbon may result in the risk of secondary contamination and a high cost (Ling et al., 2021). Therefore, effective and sustainable strategies to enhance heterotrophic denitrification in the treatment of wastewater with a low C/N ratio are needed.

Accumulating approaches have been developed for the nitrate removal from the low C/N ratio wastewater, such as the system coupled with iron-based chemical reduction and autotrophic denitrification (Liu et al., 2020), the heterotrophic denitrification system amended with redox-active biochar (Wu et al., 2019), the fungal pellets immobilized bacterial bioreactor (Zheng et al., 2021), and the utilization of biodegradable and inert carriers in the sequencing batch reactors (Huang et al., 2020). These approaches are focused on generating more electron donors to enhance nitrate removal capability or providing carriers for microorganisms to create a suitable environment. In addition, some heterotrophic denitrifying bacteria that can achieve efficient denitrification at low C/N ratio have been reported, such as *Acinetobacter* sp., *Comamonas* sp., and *Pseudomonas* sp. (Zhang S. et al., 2020; Chen et al., 2021; Fan et al., 2021). Compared with the abovementioned approaches, these denitrifying bacteria can not only effectively and readily remove nitrate from low C/N ratio wastewater without the utilization of complex engineering but also provide the functional microorganisms possessing excellent nitrate removal ability for the wastewater treatment systems. Among the denitrifying bacteria, *Pseudomonas* is the dominant bacterial genus in activated sludge and biofilm reactors (Deng et al., 2018; Zhang et al., 2019). Moreover, several *Pseudomonas* strains possess the denitrification ability and other functions, such as phosphorus removal ability and polyhydroxybutyrate-degrading ability (Di et al., 2019; Li et al., 2021). Therefore, *Pseudomonas* strains would outcompete other bacteria in the practical application.

In this study, a novel denitrifying bacterium, designated JM10B5a^T^, was isolated from the pond water for juvenile *Litopenaeus vannamei*. The phylogenetic, genomic, physiological, and biochemical analyses illustrated that strain JM10B5a^T^ represented a novel species of the genus *Pseudomonas*, for which the name *Pseudomonas oligotrophica* sp. nov. was proposed. This strain performed excellent capability for denitrification under the low C/N ratio condition. Genomic information revealed that the functional genes of *napA, narG, nirS, norB*, and *nosZ* encoding the enzymatic repertoire for completely denitrification were identified in strain JM10B5a^T^. Our findings demonstrated that strain JM10B5a^T^ could be a promising candidate for treating wastewater with a low C/N ratio.

## Materials and Methods

### Bacterial Strains

Strain JM10B5a^T^ was isolated from pond water for juvenile *Litopenaeus vannamei* collected from Jiangmen city, Guangdong Province, P. R. China (N 21° 56' 31“; E 112° 46' 16”). To isolate the aerobic denitrifying bacteria, the denitrification screening medium (DSM) was utilized. DSM was formulated as follows (per liter): sodium succinate 0.25 g, sodium citrate dihydrate 0.25 g, Na_2_HPO_4_ 1.0 g, KH_2_PO_4_ 1.0 g, NaNO_2_ 0.069 g, KNO_3_ 0.1 g, (NH_4_)_2_SO_4_ 0.066 g, MgSO_4_·7H_2_O 0.2 g, 2.0 ml of trace element solution (TES) and 1.0 ml of mixed carbon source solution (CSS), pH 7.3, and the solid plate contained the DSM supplemented with 15.0 g/L agar. MgSO_4_·7H_2_O, TES, and CSS were all added to the autoclaved DSM after filtering using 0.22-μm filter membrane. The TES contained (per liter) EDTA-Na 10.0 g, ZnSO_4_·7H_2_O 0.5 g, MnCl_2_·4H_2_O 0.4 g, CoCl_2_·2H_2_O 0.5 g, CuSO_4_·5H_2_O 0.2 g, CaCl_2_ 5.5 g, FeSO_4_·7H_2_O 1.1 g, and NaMoO_4_·2H_2_O 0.4 g. The CSS was described in the previous work (Zhang M. et al., 2020) and contained (per liter) D-glucose 13.8 g, D-fructose 13.8 g, D-lactose 13.8 g, sodium acetate 19.0 g, 90% lactic acid 12.8 ml, mannitol 14.0 g, ethyl alcohol 14.0 ml, glycerin 12.6 ml, sodium benzoate 9.6 g, and salicylic acid 9.2 g. Serial dilutions of 10^−1^ to 10^−4^ of pond water were made, and 0.1 ml of the 10^−2^, 10^−3^, and 10^−4^ dilutions was spread on DSM agar plates, respectively. Then, these plates were incubated at 30°C for 5 days. Strain JM10B5a^T^ was isolated and stored at −80°C in the nutrient broth medium (NB) supplemented with 20% (v/v) glycerol.

*P. stutzeri* CGMCC 1.1803^T^ and *P. balearica* CCUG 44487^T^ were obtained from China General Microbiological Culture Collection Center (CGMCC) and Culture Collection University of Gothenburg (CCUG), respectively. These two type strains were used as the related strains for phenotypic, chemotaxonomic, and genetic analyses.

### Phylogenetic Analysis

Genomic DNA of strains JM10B5a^T^ was extracted from fresh cells, and the 16S rRNA gene sequence was amplified using the universal primers (27F/1492R) as described previously (Zhang et al., 2021). Sequencing was performed by GENEWIZ, Inc. (Suzhou, China). The BLAST algorithm^2^ and EzBioCloud database^3^ were used to search for similar sequences (Yoon et al., 2017). Pairwise identities of 16S rRNA gene sequences were calculated using the software DNAMAN version 8. Multiple alignments of the 16S rRNA sequences were performed using the software MAFFT version 7.037 under the L-INS-i iterative refinement (Katoh and Standley, 2014). The phylogenetic tree was reconstructed using the software IQ-TREE version 2.1.2 with the maximum likelihood (ML) method under the TN+F+I+G4 nucleotide substitution model (Felsenstein, 1981; Kalyaanamoorthy et al., 2017; Minh et al., 2020). Support for the inferred ML tree was inferred by the ultrafast bootstrapping with 1,000 replicates (Diep Thi et al., 2018). The visualization and annotation of the resulting phylogenetic tree were performed using the software MEGA version X (Kumar et al., 2018).

### Morphological Observations and Analyses of Physiological Characteristics

Cells and colonies of strain JM10B5a^T^ cultivated on the nutrient broth agar medium (NA) at 30°C for 48 h were observed by a transmission electron microscope (H7650, Hitachi) and naked eyes, respectively. Oxidase activity was determined using oxidase testing strips (HKM), and catalase activity was detected by bubble production after the addition of 3.0% H_2_O_2_ (v/v) solution. Growth under an anaerobic environment was determined after 5 days of incubation on NA at 30°C in an anaerobic pouch (MGC, Mitsubishi). Hydrolysis of casein, starch, tyrosine, and Tweens 20, 40, and 80 was investigated according to the protocols described by Lanyi (1987) and Tindall et al. (2007). Cellular motility was examined using the hanging-drop method (Bernardet et al., 2002).

The temperature range for growth was measured on NA at 4, 10, 15, 20, 25, 30, 37, 40, 45, and 50°C, respectively. The pH range for growth was determined after incubation at 30°C and 180 rpm in the modified NB medium with appropriate biological buffers (50 mM): sodium citrate buffer (pH 5.0, 5.5, and 6.0), HEPES buffer (pH 6.5, 7.0, and 7.5), Tris buffer (pH 8.0, 8.5, and 9.0), and Na_2_CO_3_/NaHCO_3_ (pH 9.5, 10.0, 11.0, and 12.0). The pH of the medium was adjusted by adding 1.0 M HCl or 1.0 M NaOH before autoclaving. The NaCl tolerance for growth was examined in the modified NB medium (without NaCl) with different NaCl concentrations (w/v, 0, 0.5, 1.0, 2.0, 3.0, 4.0, 5.0, 6.0, 7.0, 8.0, 9.0, and 10.0 %) at 30°C and 180 rpm. After incubation for 72 h, the bacterial growth was estimated by the spectrophotometric measurement of cell density (OD_600_).

Additional biochemical characteristics such as the enzyme activities, acid production by fermentation, and utilization of carbon sources were carried out using API ZYM and 20NE kits (bioMérieux) and Biolog GEN III MicroPlate (Biolog) according to the manufacturers' instructions. The API strips and Biolog results were recorded every 24 h after incubation at 30°C until all reactions were steady. All API and Biolog tests were performed in duplicate under the consistent condition.

### Analyses of Chemotaxonomic Characteristics

For fatty acid composition assay, the exponentially growing cells of strain JM10B5a^T^ were harvested. Fatty acids were saponified, methylated, and extracted using the standard protocol of the Sherlock Microbial Identification System (MIDI). The prepared fatty acids were analyzed by gas chromatography (model 7890A; Agilent) using the Microbial Identification software package with the Sherlock MIDI 6.1 system and the Sherlock Aerobic Bacterial Database (TSBA 6.1).

Polar lipids were extracted using the chloroform/methanol system and separated by two-dimensional thin-layer chromatography (TLC, Silica gel 60 F254, Merck) (Minnikin et al., 1984). The plates dotted with samples were subjected to two-dimensional development with the first solvent of chloroform–methanol–water (65:25:4, v/v) and the second solvent of chloroform–methanol–acetic acid–water (80:12:15:4, v/v). Total lipids and specific functional groups were detected using the different spray staining reagents on separate TLC plates: 10% ethanolic molybdophosphoric acid, ninhydrin, molybdenum blue, and α-naphthol–sulfuric acid. The quinone was extracted from the freeze-dried cells and determined using high-performance liquid chromatography (Agilent 1200; ODS 250 × 4.6 mm × 5.0 μm; flowing phase, methanol–isopropanol, 2:1; 1.0 ml/min) according to the methods described by Collins and Jones (1981).

### Genome Sequencing and Function Analysis

The genomic DNA of strain JM10B5a^T^ was extracted using the HiPure Bacterial DNA Kit (Magen Biotech, Guangzhou) according to the manufacturer's instruction. The genome was sequenced using the Illumina NovaSeq PE150 platform in Shanghai Majorbio Bio-Pharm Technology Co., Ltd. (Shanghai, China). Raw reads were filtered and then *de novo* assembled using the software SPAdes version 3.14.1 under “–careful” mode and with a k-mer value of 127 (Bankevich et al., 2012). Available genomes of the related type strains *P. stutzeri* CGMCC 1.1803^T^ and *P. balearica* CCUG 44487^T^ were obtained from the GenBank database with the numbers CP002881 and CP007511, respectively. The genome of strain JM10B5a^T^ was in draft status and was quality-checked using the software CheckM version 1.1.2 (Parks et al., 2015). Overall genome relatedness indices (OGRIs) including digital DNA–DNA hybridization values (dDDH) and average nucleotide identity values (ANI) were estimated using the genome-to-genome distance calculator version 2.1 online service with the recommended formula 2^4^ and the software FastANI version 1.31, respectively (Meier-Kolthoff et al., 2013; Jain et al., 2018). The UBCG pipeline which could provide an accurate phylogenomic tree for the taxonomic purpose was used (Chun et al., 2018; Na et al., 2018). The genomes of strain JM10B5a^T^ and the related type strains were annotated using the software Prokka version 1.13 (Seemann, 2014) and Rapid using Subsystem Technology (RAST) version 2.0 with the default parameters (Overbeek et al., 2014).

### Assessment of Nitrogen Removal Characteristics

The results of the API 20NE test preliminarily indicated that strain JM10B5a^T^ was capable of reducing nitrate and nitrite. Furthermore, the genome annotation revealed that strain JM10B5a^T^ possessed the functional genes including *napA, narG, nirS, norB*, and *nosZ* that participated in the denitrification process. These results suggested that strain JM10B5a^T^ had the potential capability of complete denitrification. To investigate the optimum carbon source of denitrification for strain JM10B5a^T^, the carbon source was replaced in the DSM-1 (DSM with KNO_3_ 0.36 g/L as the sole nitrogen source) or DSM-2 (DSM with NaNO_2_ 0.25 g/L as the sole nitrogen source) by sodium acetate, sodium succinate, sodium citrate, glucose, sucrose, and starch, respectively, with the C/N ratio of 10 (the molar mass of carbon to the molar mass of nitrogen). Furthermore, the initial C/N ratios of 2, 3, 4, and 5 were controlled by changing the addition amount of the optimum carbon source in the DSM-1. Before the experiments, strain JM10B5a^T^ was incubated in NB at 30°C and 180 rpm for 24 h. The suspension was centrifuged and then washed 3 times with sterile physiological saline to remove the residual medium. The biomass was resuspended in sterile water with the initial OD_600_ adjusting to 1.0. The bacterial suspension was inoculated into a conical flask containing sterile medium with the inoculum amount of 4.0% and incubated at 30°C statically. Samples taken from flasks after 48 h of incubation were used for determining the OD_600_ and chemical analyses. The medium without bacterial inoculation was used as control treatment. All the experiments were performed in biological quadruplicate.

### Analytical Methods and Statistical Analyses

The concentrations of ${\text{NO}}_{2}^{-}$-N and ${\text{NO}}_{3}^{-}$-N were measured using *N*-(1-naphthyl) ethylenediamine dihydrochloride and UV spectrophotometry, respectively, according to the standard analytical procedures (Association et al., 2005). All the data were presented as mean and standard error (SE). One-way ANOVA (multiple range test) was performed with Tukey's HSD test (*P* < 0.05) using SPSS Statistics 19.

## Results and Discussion

### 16S rRNA Gene Sequence Analyses and Chemotaxonomic Characterization

**Figure 1:**
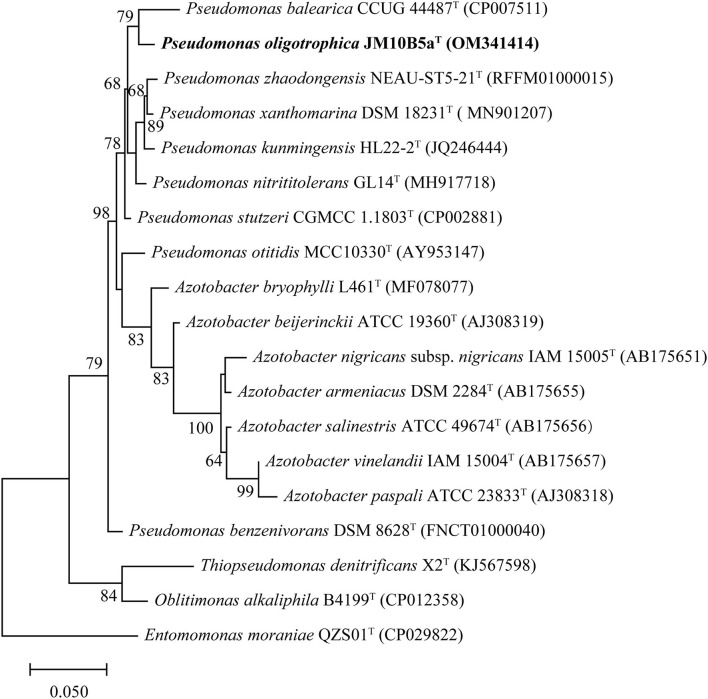
ML tree based on 16S rRNA gene sequences of strain JM10B5a^T^ and the related type strains of genus *Pseudomonas*. Type strain *Entomomonas moraniae* QZS01^T^ was used as an outgroup. There were a total of 1,303 positions used to construct the phylogenetic tree. Bootstrap values higher than 60% were shown at branch points. Bar, 0.05 represents the number of substitutions per site.

The almost complete 16S rRNA gene sequence of strain JM10B5a^T^ obtained by amplification (1,375 bp) was included in the complete 16S rRNA gene sequence assembled from genomic sequences (1,537 bp). The sequence comparison showed that strain JM10B5a^T^ fell into the genus *Pseudomonas* and shared the highest similarity with *P. stutzeri* CGMCC 1.1803^T^ (98.0%). All the other type strains showed similarities lower than 98.0% with strain JM10B5a^T^. Based on phylogenetic analysis using the ML algorithm, strain JM10B5a^T^ was stably located in the genus *Pseudomonas* and formed a clade with *P. balearica* CCUG 44487^T^ (the similarity to JM10B5a^T^ was 97.0%) at the 79.0% bootstrap confidence level (Figure 1). The similarity and phylogenetic analysis based on the 16S rRNA gene sequences indicated that strain JM10B5a^T^ should represent a novel member of the genus *Pseudomonas*. Therefore, the type strains *P. stutzeri* CGMCC 1.1803^T^ and *P. balearica* CCUG 44487^T^ were purchased from the culture collection centers and used as references for further comparisons of phenotypic and chemotaxonomic characteristics.

The predominant cellular fatty acids (>10%) of strain JM10B5a^T^ were C_16:0_ (22.0%), summed feature 3 (C_16:1_ ω6*c* and/or C_16:1_ ω7*c*, 21.3%), and summed feature 8 (C_18:1_ ω6*c* and/or C_18:1_ ω7*c*, 29.1%), which were also detected in other *Pseudomonas* species (Mamtimin et al., 2021). Comparative fatty acid profiles between strain JM10B5a^T^ and the two related type strains are shown in Table 1. The fatty acid profile of JM10B5a^T^ was similar to that of other related strains, although there were minor quantitative differences observed. The predominant respiratory quinone of strain JM10B5a^T^ was ubiquinone-9 (Q-9) which was consistent with that of other members of the genus *Pseudomonas* (Zou et al., 2019; Mamtimin et al., 2021). The major polar lipids were phosphatidylethanolamine (PE), phosphatidylglycerol (PG), and diphosphatidylglycerol (DPG), which were consistent with the previously published data for *Pseudomonas* species (Zou et al., 2019; Li et al., 2020). In addition, minor amounts of two unidentified aminophospholipids (APLs) and three unidentified phospholipids (PLs) were also present (Supplementary Figure 1).

**Table 1:** Cellular fatty acid profiles of strain JM10B5a^T^ and the closely related type strains of genus *Pseudomonas*.

**Fatty acid**	**JM10B5a^**T**^**	***P. stutzeri* CGMCC 1.1803^**T**^**	***P. balearica* CCUG 44487^**T**^**
**Straight-chain saturated**
C_12:0_	8.3	8.1	8.3
C_14:0_	1.4	1.0	0.9
C_16:0_	22.0	20.8	23.1
**Hydroxy**
C_10:0_ 3-OH	2.8	2.8	3.2
C_12:0_ 3-OH	4.0	3.8	4.1
**Branched saturated**
C_17:0_ cyclo	5.2	2.0	6.0
iso C_17:0_	TR	TR	0.8
**Unsaturated**
C_19:0_ cyclo ω8c	4.2	2.3	5.5
Summed Feature 3^*^	21.3	22.8	18.9
Summed Feature 8^*^	29.1	34.1	27.4

*All data were obtained from this study. Constituents <0.5% in three strains were not shown. TR, trace (<0.5%)*.

* *Summed features contain two or more fatty acids that cannot be separated by the MIDI system; summed feature 3 comprises C_16:1_ ω6c and/or C_16:1_ ω7c; summed feature 8 comprises C_18:1_ ω6c and/or C_18:1_ ω7c*.

### Morphological Observations and Physiological Characterization

The morphological features of strain JM10B5a^T^ were studied on NA medium and formed irregular, filmy sheet, non-transparent, and ecru white colonies after 48 h of incubation at 30°C (Supplementary Figure 2A). Cells of strain JM10B5a^T^ were observed to be gram-stain-negative, aerobic, rod-shaped (0.6–0.7 × 1.4–1.5 μm), facultative anaerobic, and motile with a single polar flagellum (Supplementary Figure 2B). Growth was determined at 10–45°C (optimum, 25–30°C), at pH 5.5–11.0 (optimum, 6.0), and in 0–4.0% (w/v) NaCl (optimum, 0.5–2.0%). The activities of catalase and oxidase were positive, and the further details differentiating strain JM10B5a^T^ from the two related type strains are shown in Table 2. For instance, strain JM10B5a^T^ could not grow in the NB with a NaCl concentration of 5.0 while the two related type strains could grow; the optimum pH of strain JM10B5a^T^ was 6.0, which was inconsistent with that of *P. stutzeri* CGMCC 1.1803^T^ and *P. balearica* CCUG 44487^T^ of 6.5–7.5 and 8.5–9.5, respectively; the growth of strain JM10B5a^T^ was weaker than that of the two related type strains at 45°C. Besides, stain JM10B5a^T^ could be distinguished from the reference type strains by positive for adipic acid, trisodium citrate, esterase (C4), and lipase (C14) in the API 20NE and ZYM tests; and positive for D-galacturonic acid, D-glucuronic acid, gentiobiose, L-arginine, L-aspartic acid, L-galactonic acid lactone, mucic acid, quinic acid, α-hydroxybutyric acid, and γ-aminobutyric acid in the Biolog GNE III MicroPlate system. Importantly, strain JM10B5a^T^ exhibited the capability for complete denitrification that liberated copious amounts of nitrogen gas from nitrate. The denitrification characteristic of this strain was consistent with that of *P. stutzeri* and *P. balearica* strains (Bennasar et al., 1996; Li et al., 2021).

**Table 2:** Differential characteristics of strain JM10B5a^T^ and the closely related type strains of genus *Pseudomonas*.

**Characteristic**	**JM10B5a^**T**^**	***P. stutzeri* CGMCC 1.1803^**T**^**	***P. balearica* CCUG 44487^**T**^**
**Growth**		
NaCl concentration (optimum, w/v)	0–4.0 (0.5–2.0)	0–5.0 (0.5)	0–5.0 (1.0)
pH range (optimum)	5.5–11.0 (6.0)	6.0–11.0 (8.5–9.5)	5.5–11.0 (6.5–7.5)
Temperature (optimum, °C)	10–45 (25–30)	10–45 (30–37)	10–45 (30–37)
45°C	w	+	+
**API ZYM and 20NE tests**			
Adipic acid	+	+	–
Trisodium citrate	+	+	–
Esterase (C4)	+	–	–
Lipase (C14)	+	+	–
**Biolog (GEN III) tests**		
Dextrin	–	+	+
D-Galacturonic acid	+	–	–
D-Glucuronic acid	+	w	–
D-Maltose	–	+	+
D-Mannitol	–	+	–
D-Saccharic acid	+	–	–
Formic acid	–	+	–
Gentiobiose	+	–	–
L-Arginine	+	–	–
L-Aspartic acid	+	–	+
L-Galactonic acid lactone	+	–	–
L-Pyroglutamic acid	–	+	–
Mucic acid	+	–	–
Quinic acid	+	–	–
α-Hydroxy-butyric acid	+	–	+
γ-Amino butyric acid	+	–	–
**G** **+** **C content (%)**	67.2	63.6^§^	64.7^§^
**Genome size (Mb)**	4.0	4.5^§^	4.4^§^

*All data were obtained from this study unless indicated otherwise*.

§ *Data from draft genomes in the NCBI genome database*.

*+, positive; –, negative; w, weakly positive*.

### Genome Data and Comparative Genomic Analysis

**Figure 2:**
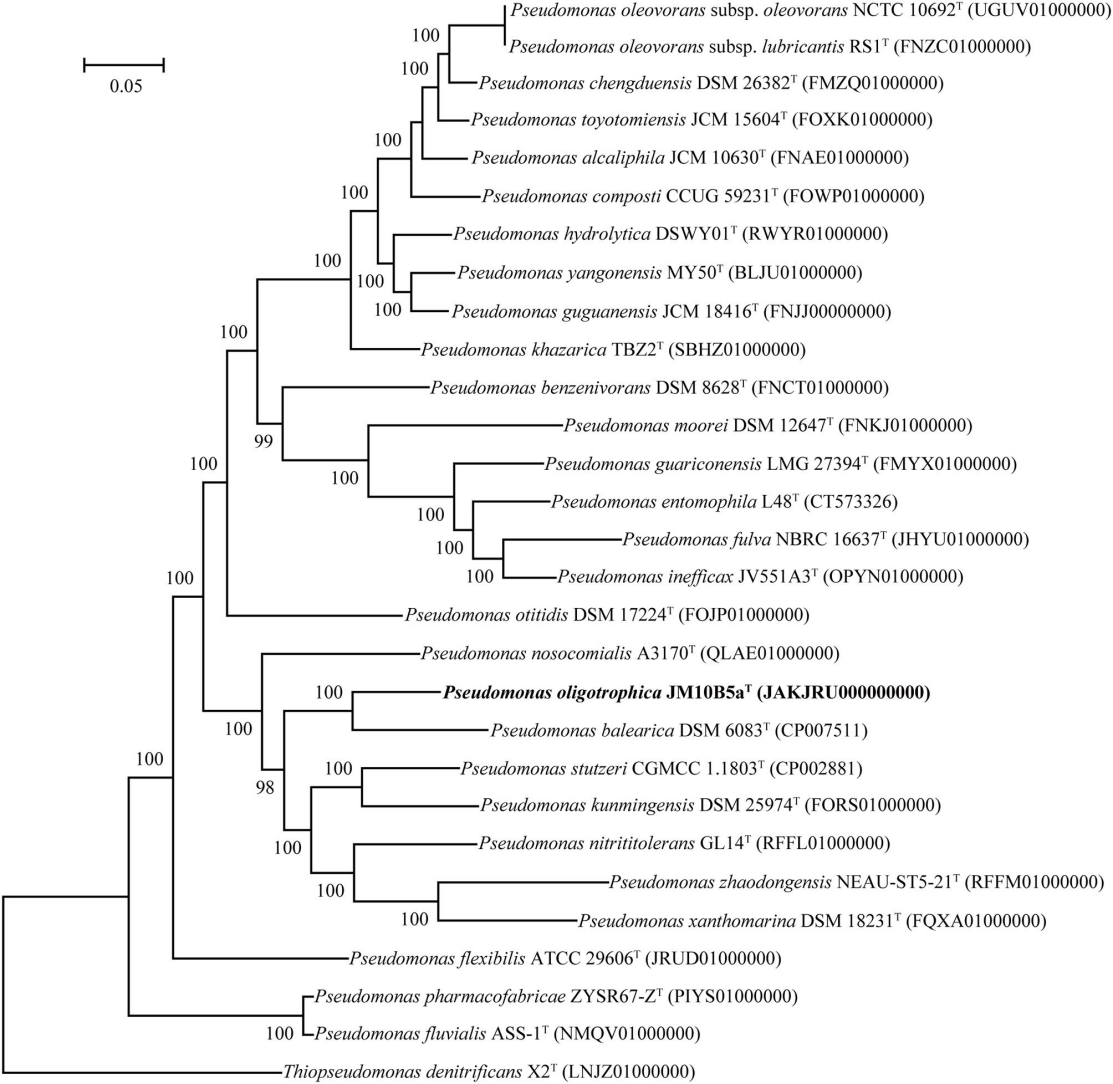
Phylogenomic tree based on 92 bacterial core gene sets of strain JM10B5a^T^ and the related type strains of genus *Pseudomonas*. Type strain *Thiopseudomonas denitrificans* X2^T^ was used as an outgroup. Bootstrap values higher than 70% were shown at branch points. Bar, 0.05 represents the number of substations per site.

The draft genome of strain JM10B5a^T^ was assembled into 20 contigs (>500 bp) with a genome size of 3,978,222 bp, and the estimated completeness and contamination were 100 and 0.1%, respectively, indicating that this genome was high quality according to the standards (Parks et al., 2015). Genomes of strains *P. stutzeri* CGMCC 1.1803^T^ and *P. balearica* CCUG 44487^T^ were obtained from the GenBank database with the numbers CP002881 and CP007511, respectively. Based on the Prokka annotation, a total of 3,744 genes, 3,679 protein-coding sequences (CDS), 60 tRNA genes, and 3 rRNA genes were found in the genome of strain JM10B5a^T^. The genomic DNA G + C content of JM10B5a^T^ was 67.2%. Compared with the genomes of related type strains, JM10B5a^T^ had ANI values of 78.9–88.1% and dDDH values of 20.8–30.8% (Supplementary Table 1), which were all below 95.0 and 70% cutoff commonly used to define a bacterial species, respectively (Stackebrandt and Goebel, 1994; Richter and Rossello-Mora, 2009). To further determine the taxonomic position of strain JM10B5a^T^, the phylogenomic tree based on the 92 bacterial core gene sets was reconstructed (Na et al., 2018). As shown in Figure 2, strain JM10B5a^T^ and *P. balearica* CCUG 44487^T^ formed a clade with 100% bootstrap value, which was consistent with the phylogenetic tree based on the 16S rRNA gene sequences. Therefore, the comprehensive analyses of OGRIs and phylogenomic tree further indicated that strain JM10B5a^T^ should represent a novel species within the genus of *Pseudomonas*.

**Figure 3:**
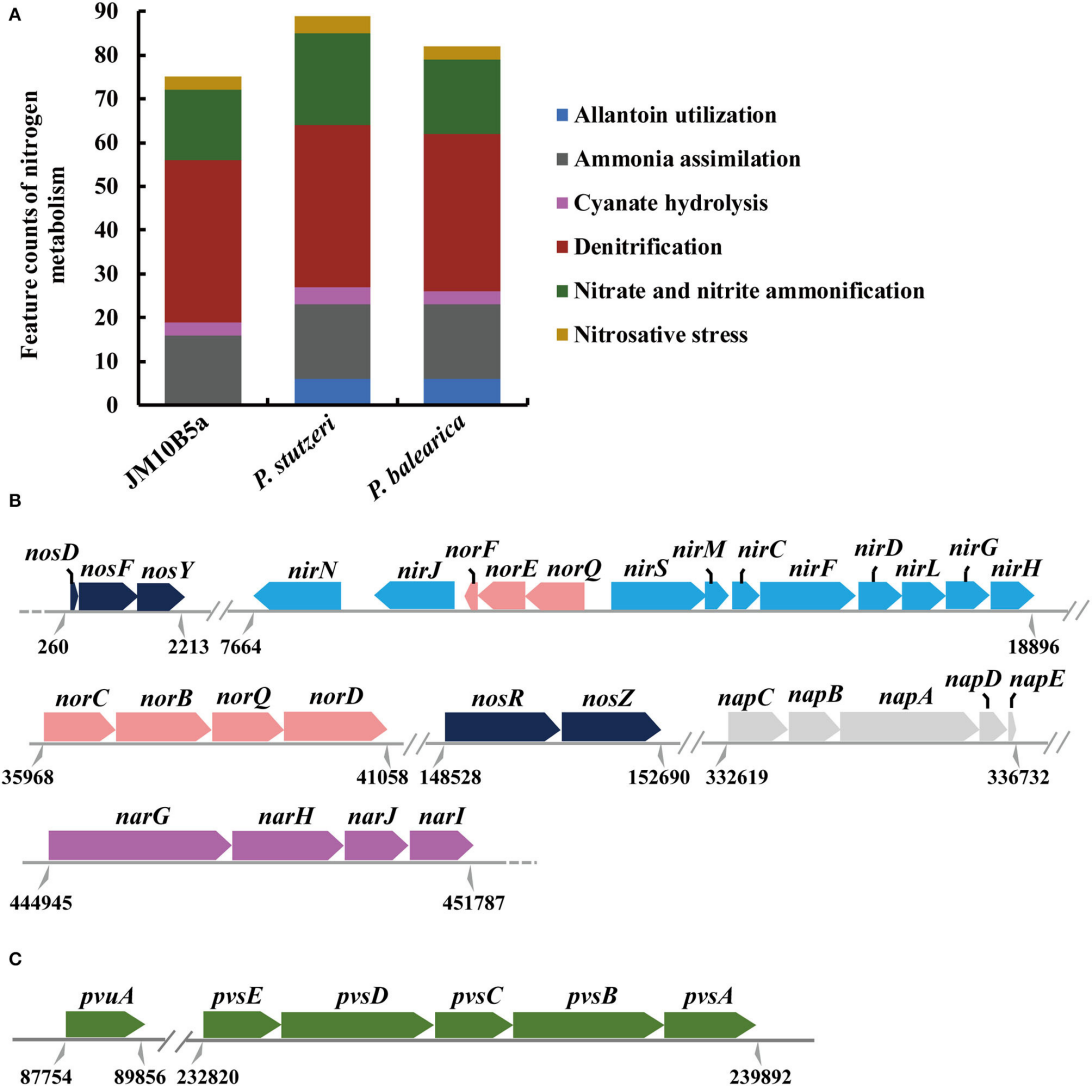
Genomic analyses based on the RAST annotation. **(A)** Comparative analyses of the functional genes assigned to nitrogen metabolisms of strain JM10B5a^T^ and the related type strains of genus *Pseudomonas*. **(B)** Relative arrangements of the denitrification genes on the draft genome of strain JM10B5a^T^. **(C)** The biosynthetic gene cluster for vibrioferrin on the draft genome of strain JM10B5a^T^. Parallel double lines indicate a break in locus organization among scaffolds, and dotted black lines indicate where unrelated continuity loci are not shown. Numbers below the line symbolize the locations.

According to the subsystem category distribution of RAST annotation, there were 190, 128, 402, and 336 genes associated with “RNA metabolism,” “DNA metabolism,” “amino acid and derivatives,” and “carbohydrates,” respectively, which might be important to the bacterial growth. In addition, 75 genes were identified in the nitrogen metabolism, including the functional genes related to denitrification and assimilation of nitrite and nitrate (Figures 3A,B). Nitrate reductase catalyzing the reduction in nitrate to nitrite plays an important role in the nitrogen cycle (Kuypers et al., 2018). Previous studies have shown that bacterial dissimilatory nitrate reduction could be catalyzed by two different enzymes: a membrane-bound nitrate reductase (NAR, the catalytic subunit NarG encoded by *narG*) and periplasmic nitrate reductase (NAP, the catalytic subunit NapA encoded by *napA*) (Moreno-Vivian et al., 1999). Genes *narG* and *napA* were both contained in strain JM10B5a^T^, which was consistent with *Pseudomonas* sp. JQ-H3 and *Paracoccus denitrificans* (Moreno-Vivian et al., 1999; Wang et al., 2019). The dissimilatory reduction in nitrite to nitric oxide can be catalyzed by two unrelated enzymes: a cytochrome cd1 nitrite reductase (cd1-NIR, encoded by *nirS*) or a Cu-containing nitrite reductase (Cu-NIR, encoded by *nirK*), which were widespread among bacteria and archaea (Kuypers et al., 2018). Gene *nirS* rather than *nirK* was identified in strain JM10B5a^T^, which was consistent with *Pseudomonas* sp. JQ-H3, *P. bauzanensis* DN13-1, and *P. stutzeri* T13 (Wang et al., 2019; Zhang M. et al., 2020; Feng et al., 2021). In addition, genes *norB* encoding for nitric oxide reductase (NorB, the key enzyme for reducing nitric oxide to nitrous oxide) and *nosZ* encoding for nitrous oxide reductase (NosZ, the key enzyme for reducing nitrous oxide to nitrogen gas) were also identified. Therefore, it was speculated that strain JM10B5a^T^ could perform the complete denitrification pathway: ${\text{NO}}_{3}^{-}$-N → ${\text{NO}}_{2}^{-}$-N → NO → N_2_O → N_2_. Hence, strain JM10B5a^T^ was a novel denitrifying bacterium.

In addition, comparative genomic analyses showed that strain JM10B5a^T^ encoded 126 and 147 functional genes more than *P. balearica* CCUG 44487^T^ and *P. stutzeri* CGMCC 1.1803^T^, respectively, and 67 genes of them were the shared differential genes (Supplementary Table 2). These genes were related to type I secretion system for aggregation, utilization of alkylphosphonate and tricarballylate, histidine ABC transporter, LysR family transcriptional regulator, and many other proteins. Most of these proteins were mainly affiliated with dehydratase, transferase, and biosynthesis of cofactors and vitamins. The genes encoding arginase and galactarate dehydratase made strain JM10B5a^T^ performing the positive for L-arginine and mucic acid (Table 2). Moreover, strain JM10B5a^T^ was found to harbor the complete operon *pvsABCDE* and vibrioferrin receptor gene *pvuA* (Figure 3C) that are required for the production of a siderophore vibrioferrin. This siderophore was shown to be responsible for vibrioferrin-mediated iron uptake in the terrestrial bacteria *Azotobacter vinelandii, Pseudomonas* sp., and the marine bacterium *Vibrio parahaemolyticus* (Yamamoto et al., 1994; Baars et al., 2016; Stanborough et al., 2018; Sood et al., 2019). It was reported that siderophore had a very high affinity to iron with the formation of Fe-siderophore which could facilitate the transport of iron through the membrane directly (Stintzi et al., 2000). Moreover, the denitrification efficiency might be enhanced with the improvement of iron transport from extracellular to intracellular (Jiang et al., 2020). Therefore, the biosynthesis of vibrioferrin might partly explain that the strain JM10B5a^T^ performed the high denitrification efficiency under the low C/N ratio condition.

### Effect of Carbon Source on Denitrification by Strain JM10B5a^T^

**Figure 4:**
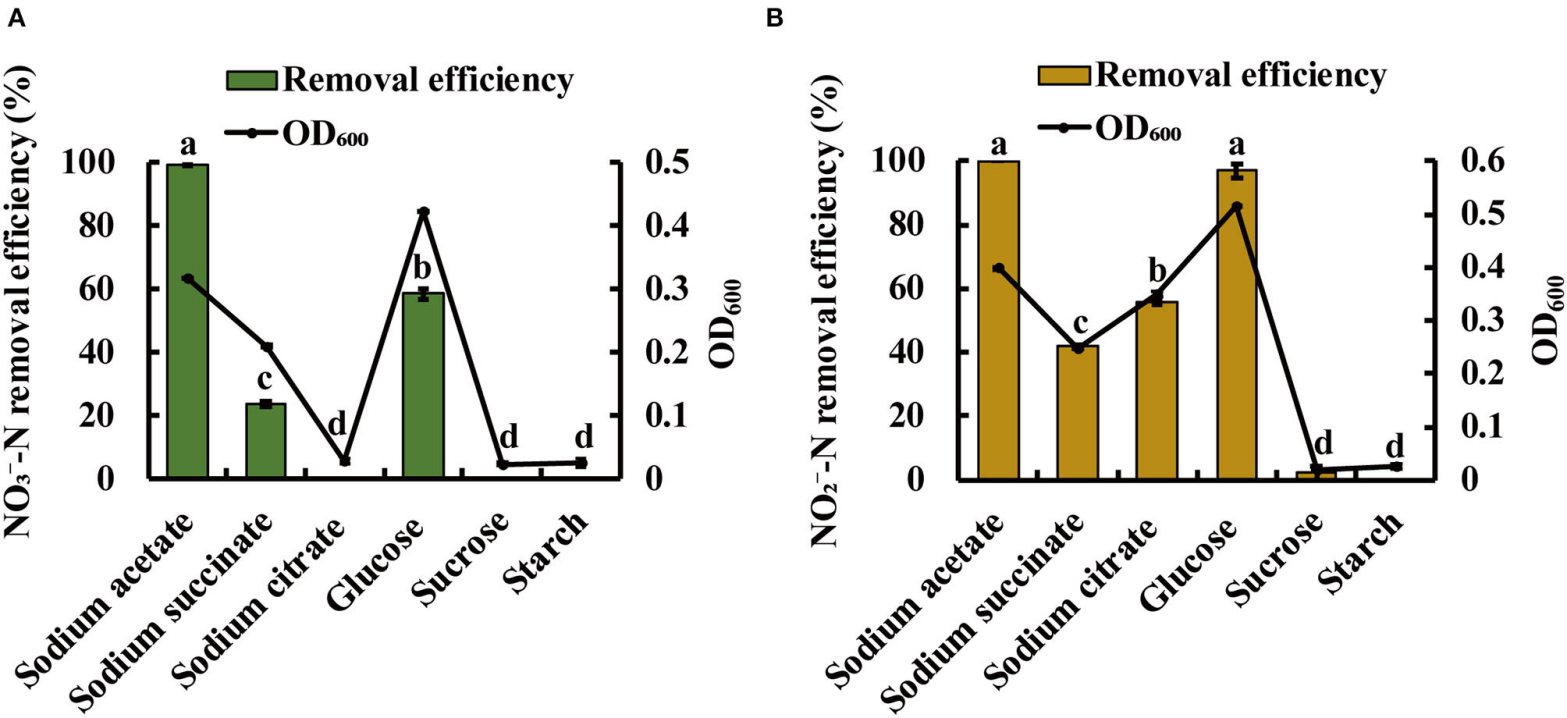
Growth (OD_600_) and ${\text{NO}}_{3}^{-}$-N and ${\text{NO}}_{2}^{-}$-N removal performance of strain JM10B5a^T^ under various common carbon sources. **(A)** ${\text{NO}}_{3}^{-}$-N as the sole nitrogen source. **(B)** ${\text{NO}}_{2}^{-}$-N as the sole nitrogen source. Values are mean ± SE (standard error) for four replicates.

Heterotrophic denitrifying bacteria require organic carbon for cell growth and as the electron donor in the denitrification process (Rajta et al., 2020). Thus, carbon source was considered to be an important factor influencing denitrification. As expected, significant differences were observed using different carbon sources. As shown in Figure 4A, the strain JM10B5a^T^ could grow well with the OD_600_ of 0.42 when glucose was used as the sole carbon source. However, it exhibited a higher ${\text{NO}}_{3}^{-}$-N removal efficiency of 99.5% when sodium acetate served as carbon source than that of 58.6% when glucose served as carbon source. As shown in Figure 4B, strain JM10B5a^T^ could grow quite well when glucose served as the carbon source (OD_600_ of 0.52), but it performed the relatively high ${\text{NO}}_{2}^{-}$-N removal of 100% when sodium acetate served as the carbon source. These results indicated that sodium acetate was the optimum carbon source for the performance of denitrification for strain JM10B5a^T^, which was consistent with *Bacillus pumilus, Arthrobacter* sp., and *Streptomyces lusitanus* (Elkarrach et al., 2021). Therefore, sodium acetate was employed in the following experiments.

### Effect of Carbon/Nitrogen Ratio on Nitrate Removal by Strain JM10B5a^T^

**Figure 5:**
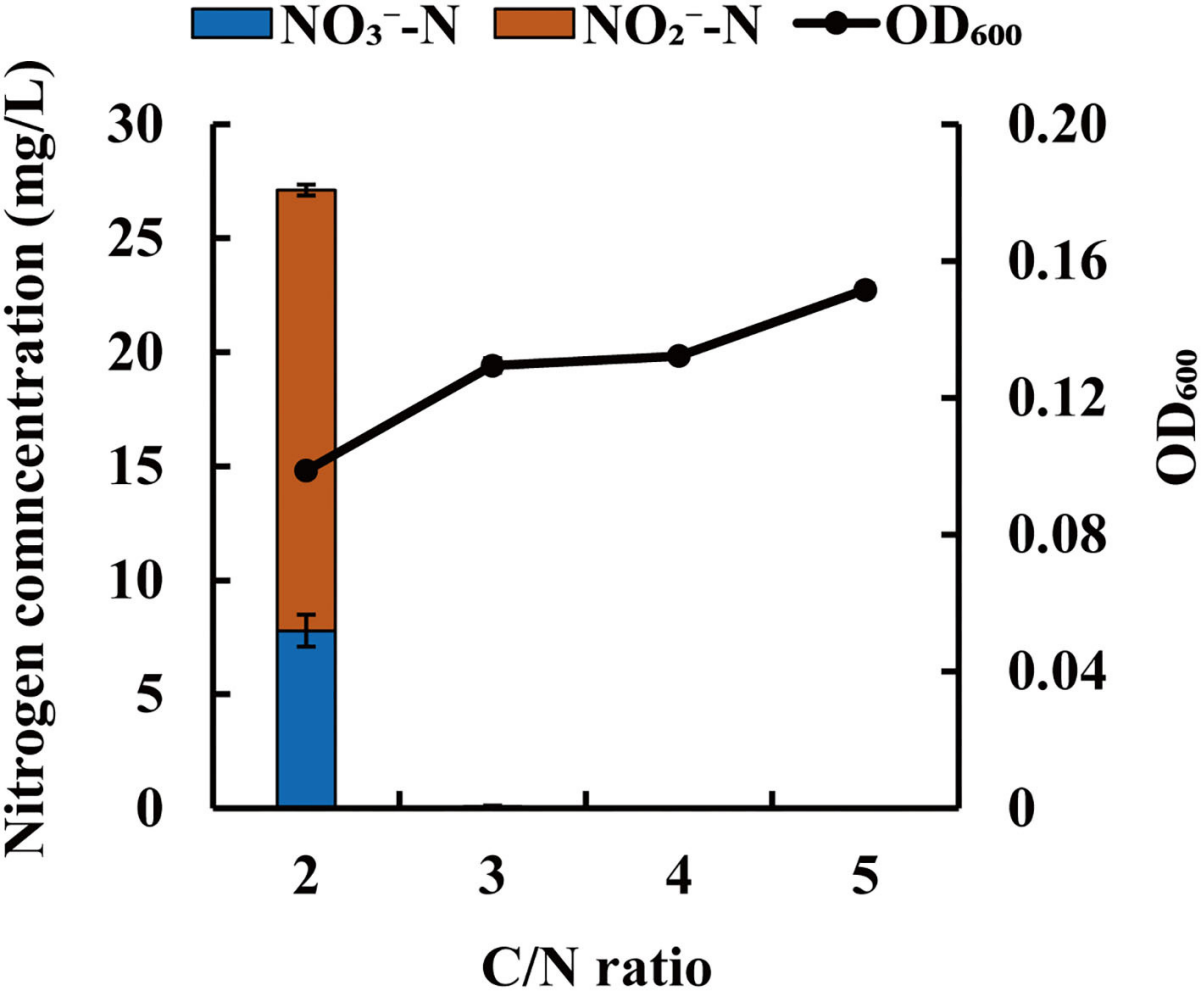
Influence of C/N ratio on ${\text{NO}}_{3}^{-}$-N removal characteristic of strain JM10B5a^T^. Values are mean ± SE (standard error) for four replicates.

C/N ratio is a measure of the electron donor to acceptor ratio in the biological denitrification process. This factor can influence the denitrification efficiency and accumulation of ${\text{NO}}_{2}^{-}$-N. To investigate the influence of C/N ratio on denitrification of strain JM10B5a^T^, the initial concentration of ${\text{NO}}_{3}^{-}$-N was fixed at ~50.0 mg/L and the different initial C/N ratios (2, 3, 4, and 5) were controlled. As shown in Figure 5, strain JM10B5a^T^ grew poorly with the OD_600_ range of 0.10–0.15 at these low C/N ratios. The ${\text{NO}}_{3}^{-}$-N was reduced completely at the C/N ratios of 3–5 which was distinctly higher than that of 84.4% at the C/N ratio of 2. Furthermore, there was no ${\text{NO}}_{2}^{-}$-N accumulation observed at the C/N ratios of 3–5, but the ${\text{NO}}_{2}^{-}$-N concentration of 19.3 mg/L was detected at the C/N ratio of 2. The low ${\text{NO}}_{3}^{-}$-N removal efficiency and ${\text{NO}}_{2}^{-}$-N accumulation at the C/N ratio of 2 were mainly due to the limited carbon source that could not provide sufficient energy for bacterial growth and electron donors for denitrification (Fan et al., 2021). These results were consistent with previous reports that denitrification efficiency could decrease under the extremely low carbon concentration (Zhao et al., 2018). Numerous studies have suggested that the optimum C/N ratios for most heterotrophic denitrifying bacteria were in the range of 8–15 (Guo et al., 2016; Liu et al., 2018; Zhao et al., 2018). For example, the C/N ratios of 10 and 15 were the most suitable for strains *P. stutzeri* XL-2 and *P. taiwanensis* J to achieve efficient nitrate removal, respectively (He et al., 2018; Zhao et al., 2018). In addition, some denitrifying bacteria could achieve complete denitrification at the relatively low C/N ratios. For example, Rout et al. (2017) used *Bacillus cereus* to remove nitrogen from domestic wastewater with 7.5 as the optimum C/N ratio; Zhang S. et al. (2020) reported that strain *Comamonas* sp. YSF15 could achieve complete denitrification at C/N of 3. Our results showed that strain JM10B5a^T^ could achieve the complete nitrate removal without accumulation of nitrite at the low carbon–nitrogen ratio of 3 (COD_Cr_/TN ratio of 2.6), indicating that this strain could be a promising candidate for treating the oligotrophic wastewater.

### Description of *Pseudomonas oligotrophica* sp. nov.

*Pseudomonas oligotrophica* (o.li.go.tro'phi.ca. Gr. adj. *oligos*, few; Gr. adj. *trophikos*, nursing, tending, or feeding; N.L. fem. adj. *oligotrophica*, eating little, referring to a bacterium living on low-nutrient media).

Cells are rods (1.4–1.5 μm in length and 0.6–0.7 μm in width), gram-stain-negative, facultative anaerobic, and motile by the polar flagellum. Colonies are irregular, filmy sheet, non-transparent, and ecru white after 48 h of incubation on NA agar at 30°C. Growth was determined at 10–45°C (optimum, 25–30°C), at pH 5.5–11.0 (optimum, 6.0), and in 0–4.0% (w/v) NaCl (optimum, 0.5–2.0%) and is positive for catalase and oxidase, and hydrolysis of Tween 20 and 60. In the API ZYM and 20NE system, it is positive for esterase (C4), esterase lipase (C8), lipase (C14), leucine arylamidase and naphtol-AS-B1-phosphoamidase, nitrate and nitrite reduction, β-glucosidase, D-glucose, D-maltose, potassium gluconate, capric acid, adipic acid, malic acid, and trisodium citrate. In the Biolog GNE III MicroPlate system, it is positive for gentiobiose, α-D-glucose, D-fructose, glycerol, L-alanine, L-arginine, L-aspartic acid, L-glutamic acid, D-galacturonic acid, L-galactonic acid lactone, D-gluconic acid, D-glucuronic acid, glucuronamide, mucic acid, quinic acid, D-saccharic acid, L-lactic acid, citric acid, α-ketoglutaric acid, D-malic acid, L-malic acid, bromosuccinic acid, γ-aminobutyric acid, α-hydroxybutyric acid, β-hydroxy-D, L-butyric acid, α-ketobutyric acid, propionic acid, and acetic acid. The major fatty acids are C_16:0_, summed feature 3 (C_16:1_ ω6*c* and/or C_16:1_ ω7*c*), and summed feature 8 (C_18:1_ ω6*c* and/or C_18:1_ ω7*c*). The major polar lipids are PE, PG, and DPG, and the predominant respiratory quinone is Q-9. The DNA G + C content of strain JM10B05a^T^ is 67.2%. Accession numbers of the complete 16S rRNA gene sequence and draft genome in DDBJ/ENA/GenBank are OM341414 and JAKJRU000000000, respectively. The type strain, JM10B5a^T^ (= GDMCC 1.2828^T^ = JCM 35033^T^), was isolated from pond water for juvenile *Litopenaeus vannamei* collected from Jiangmen city Guangdong Province, China.

## Data Availability Statement

The datasets presented in this study can be found in online repositories. The names of the repository/repositories and accession number(s) can be found in the article/Supplementary Material.

## Author Contributions

MZ, AL, and QY were involved in conceptualization and project administration. MZ involved in data curation, software, visualization, and writing—original draft. MZ and BX designed the formal analysis and methodology. AL, HZ, and BX were involved in funding acquisition. MZ, AL, QY, BX, and HZ investigated the study. HZ collected the resources and supervised the study. MZ and AL validated the study. MZ, QY, and BX were involved in writing—review and editing. All authors contributed to the article and approved the submitted version.

## Funding

This work was financially supported by the Key-Area Research and Development Program of Guangdong Province (2020B0202080005), the National Natural Science Foundation of China (32070115, 11772133), and the GDAS' Special Project of Science and Technology Development (2020GDASYL-20200301003).

## Conflict of Interest

AL was employed by Guangdong BOWOTE BioSciTech, Co. Ltd. The remaining authors declare that the research was conducted in the absence of any commercial or financial relationships that could be construed as a potential conflict of interest.

## Publisher's Note

All claims expressed in this article are solely those of the authors and do not necessarily represent those of their affiliated organizations, or those of the publisher, the editors and the reviewers. Any product that may be evaluated in this article, or claim that may be made by its manufacturer, is not guaranteed or endorsed by the publisher.

## Footnotes

^1^ https://help.ezbiocloud.net/ubcg-gene-set/

^2^ https://blast.ncbi.nlm.nih.gov/Blast.cgi

^3^ https://www.ezbiocloud.net/

^4^ http://ggdc.dsmz.de/ggdc.php/

## Abbreviations

ANI: average nucleotide identity
CCUG: Culture Collection University of Gothenburg
CDS: protein-coding sequences
CGMCC: China General Microbiological Culture Collection Center
dDDH: digital DNA–DNA hybridization
DPG: diphosphatidylglycerol
DSM: denitrification screening medium
GDMCC: Guangdong Microbial Culture Collection Center
JCM: Japan Collection of Microorganisms
ML: maximum likelihood
NA: nutrient broth agar medium
NB: nutrient broth medium
OGRIs: overall genome relatedness indices
PE: phosphatidylethanolamine
PG: phosphatidylglycerol
RAST: Rapid Annotation using Subsystem Technology
TLC: thin-layer chromatography
UBCG: up-to-date bacterial core gene.
