# Supplementary material for: Pseudomonas oligotrophica sp. nov., a Novel Denitrifying Bacterium Possessing Nitrogen Removal Capability Under Low Carbon–Nitrogen Ratio Condition
Source: Front Microbiol. 2022 May 20;13:882890. doi: 10.3389/fmicb.2022.882890 (PMC9164167; doi:10.3389/fmicb.2022.882890)
Supplement: Supplementary file 1 [file Image_1.PDF]

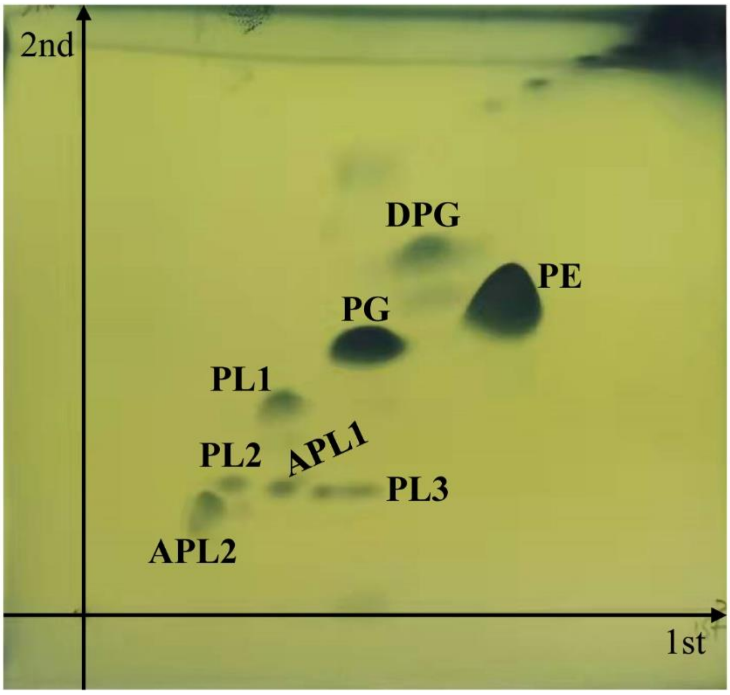

Figure 1 Two-dimensional thin-layer chromatogram of total polar lipids from strain JM10B5a^{T}.

PE, phosphatidylethanolamine; PG, phosphatidylglycerol; DPG, diphosphatidylglycerol; PL, unidentified phospholipid; APL, unidentified aminophospholipid.
