# Supplementary figures and images for: Pseudomonas oligotrophica sp. nov., a Novel Denitrifying Bacterium Possessing Nitrogen Removal Capability Under Low Carbon–Nitrogen Ratio Condition
Source: Front Microbiol. 2022 May 20;13:882890. doi: 10.3389/fmicb.2022.882890 (PMC9164167; doi:10.3389/fmicb.2022.882890)

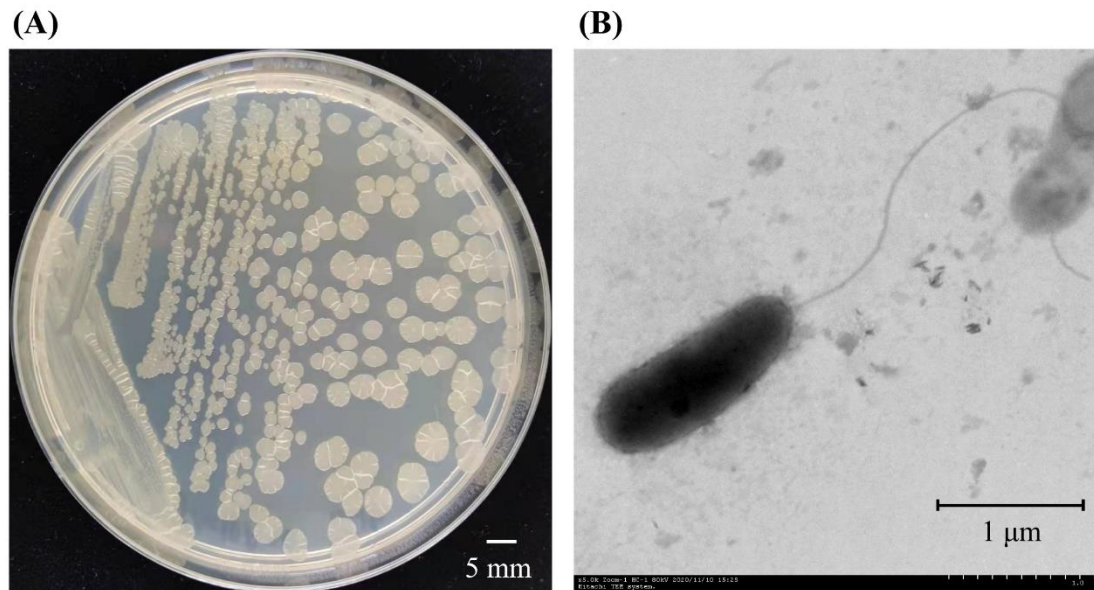

Figure 2 Colonies and cell morphology of strain JM10B5a<sup>T</sup> cultured on NA medium at 30°C for 48 h.

Supplement: Supplementary file 2 [file Image_2.PDF]
